# Supplementary material for: Complex‐centric proteome profiling by SEC‐SWATH‐MS
Source: Mol Syst Biol. 2019 Jan 14;15(1):e8438. doi: 10.15252/msb.20188438 (PMC6346213; doi:10.15252/msb.20188438)
Supplement: Supplementary file 7 — Dataset EV6 [file MSB-15-e8438-s007.zip › feature_plots_bioplex/Q8N140.pdf]

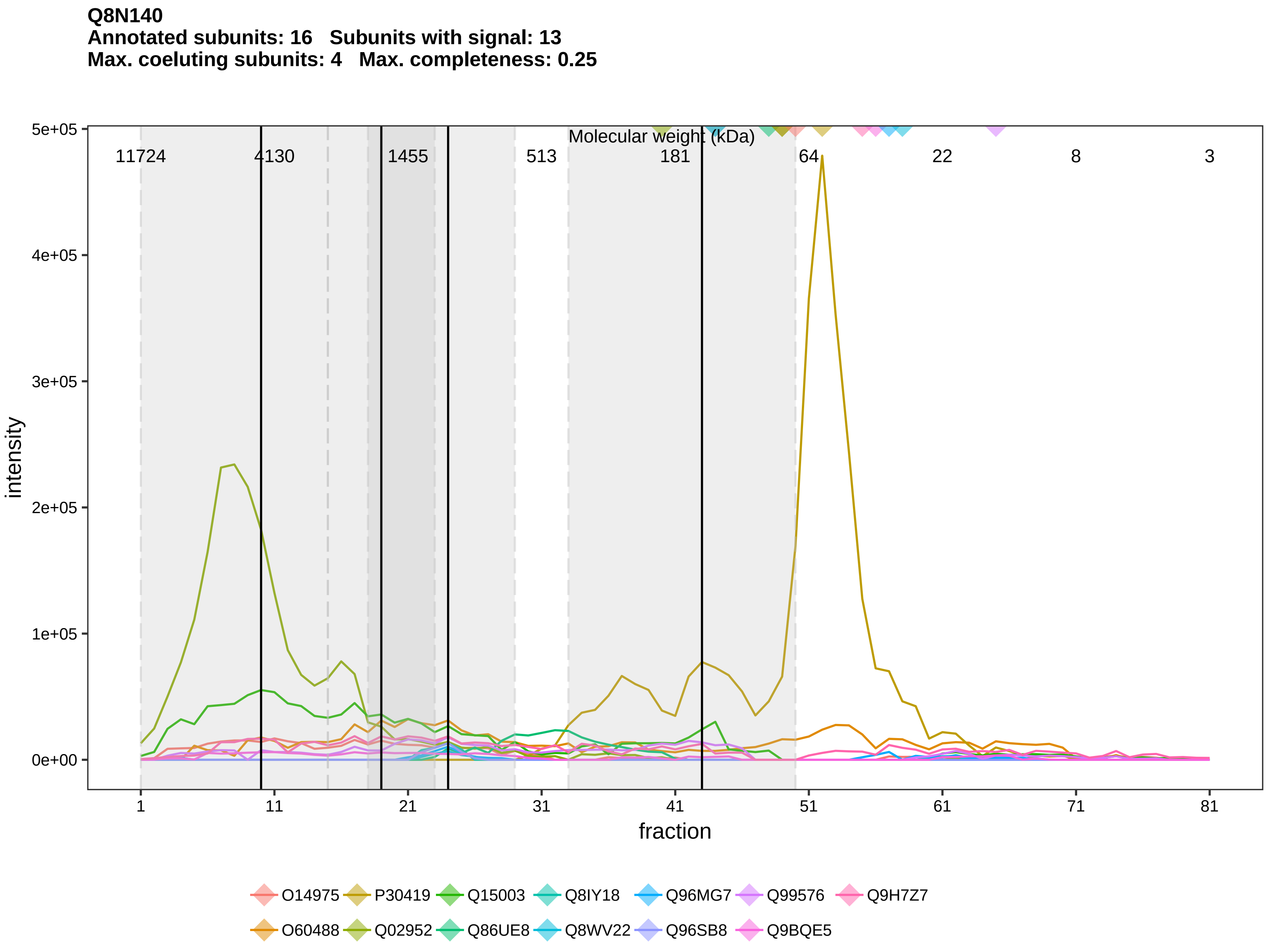
Q8N140
Annotated subunits: 16 Subunits with signal: 13
Max. coeluting subunits: 4 Max. completeness: 0.25
Molecular weight (kDa)
11724
4130
1455
513
181
64
22
8
3
intensity
5e+05
4e+05
3e+05
2e+05
1e+05
0e+00
fraction
1
11
21
31
41
51
61
71
81
O14975
P30419
Q15003
Q8IY18
Q96MG7
Q99576
Q9H7Z7
O60488
Q02952
Q86UE8
Q8WV22
Q96SB8
Q9BQE5
